# Physicians' Perspectives on the Impact of Insurance Status on Clinical Decision-Making in Saudi Arabia

**DOI:** 10.7759/cureus.53756

**Published:** 2024-02-07

**Authors:** Abdullah A Alotaibi, Khalid A Alotaibi, Ahmad N Almutairi, Anas Alsaab

**Affiliations:** 1 Family Medicine, Manzil Healthcare Services, Riyadh, SAU; 2 Internal Medicine, King Abdulaziz Medical City Riyadh, Riyadh, SAU; 3 Family Medicine, Medical Services in Saudi Royal Land Forces, Riyadh, SAU; 4 Family Medicine, King Saud Medical City, Riyadh, SAU

**Keywords:** impact, insurance status, saudi arabia, clinical decision-making, physician

## Abstract

Background

The decision-making process in clinical practice depends heavily on collaboration and information sharing. Physicians' decision-making processes are profoundly influenced by the patient's insurance status, which warrants focused investigation. Hence, this study aimed to investigate how physicians perceive the influence of insurance status on treatment options and medical interventions and to explore the extent to which physicians discuss insurance-related considerations with patients during the shared decision-making process.

Methodology

This was a cross-sectional exploratory study conducted in various healthcare facilities all over Saudi Arabia. The electronic questionnaire was the primary tool for data collection. Data were then coded, entered, and analyzed using both descriptive and inferential statistical methods.

Results

The study involved 430 physicians, primarily male (*n *= 230, 53.5%), aged 31-40 years (*n *= 215, 50%), and mostly non-Saudi (*n *= 285, 66.3%). Medical officers constituted the majority of the study population (*n *= 258, 60%), with one to five years of experience (*n *= 187, 43.5%), and engaged in private practice (*n *= 230, 70%). Concerning insurance, 287 (66.7%) physicians considered patient's insurance when discussing treatment options, while 318 (74%) physicians discussed the financial implications of different treatment options with the patients. Regarding outcomes, 373 (86.7%) physicians believed that insurance status affected patient outcomes and treatment modalities. Significant factors, such as age between 31 and 40 years (*P* < 0.001), over 10 years of clinical experience (*P* = 0.002), engagement in both governmental and private practice (*P* = 0.012), and being a medical officer (*P* = 0.005), demonstrated a high impact on the insurance status influencing clinical decision-making. Overall, recognizing the influence of insurance on decision-making is crucial for equitable healthcare.

Conclusions

More than half of the physicians demonstrated high scores indicating the impact of insurance status on the clinical decision-making process. This impact was influenced by specific physician parameters such as age, experience, specialty, and type of practice. Moreover, the financial situation and insurance status of the patients significantly affected treatment and patient outcomes.

## Introduction

Clinical decision-making is a collaborative process that hinges on the exchange of information [1]. In this process, healthcare providers disseminate the advantages and drawbacks of various treatment possibilities, while patients contribute their personal values and lifestyle choices. This amalgamation of data facilitates decisions grounded in the most relevant evidence, tailored to individual preferences and requirements. The crux of decision-making arises when several medically viable alternatives are at hand, with no universally optimal solution.

The contemporary landscape of healthcare systems is marked by a dynamic interplay between clinical decision-making and an array of external factors that collectively shape patient care [2]. Among these, influential factors, the patient’s insurance status has risen to prominence, warranting focused investigation into its impact on the decision-making processes of the physicians [3].

Particularly within the context of Saudi Arabia, a country undergoing rapid advancements in healthcare, the intricate relationship between insurance status and clinical decision-making has emerged as a subject of paramount importance [4]. This research embarks on a comprehensive exploration, aiming to unravel the nuanced layers of physicians’ perceptions concerning how insurance status intricately influences their clinical decision-making practices within the intricate tapestry of the Saudi Arabian healthcare landscape.

This study posits that the interface between insurance status and clinical decision-making is an intricate and multifaceted domain that extends far beyond monetary considerations. It signifies a juncture where the art and science of medicine converge with socioeconomic dynamics, ethical considerations, and patient-centered care. The choices physicians make, guided by their perceptions and insights, hold the potential to be influenced by a patient’s insurance status, thereby shaping the trajectory of medical interventions, diagnostic pathways, and treatment modalities [5]. Specifically, it seeks to comprehend the intricate ways in which insurance status reverberates through the corridors of clinical decision-making in Saudi Arabia’s intricate healthcare.

As Saudi Arabia forges ahead in its mission to elevate its healthcare ecosystem, the insights derived from this research hold significant implications. A comprehensive grasp of physicians’ perceptions regarding the intricate interplay between insurance status and clinical decision-making could illuminate potential areas of improvement in patient care, resource allocation, and policy formulation [3]. The findings can catalyze informed dialogues among stakeholders, fostering a healthcare system that is not only technologically advanced but also deeply attuned to the diverse needs and dynamics of its patient population.

The nexus between insurance status and clinical decision-making has garnered substantial global attention within the healthcare discourse in recent years. The research by Riley has underscored the formidable challenges that uninsured patients often encounter in accessing comprehensive medical care, exacerbating pre-existing disparities within healthcare systems [6]. This observation has illuminated the profound implications of insurance status, reaching far beyond financial implications, and reinforcing the need for equitable healthcare access.

Interestingly, within the context of Saudi Arabia, a parallel perspective has remained relatively underexplored. Al-Hanawi et al. ventured into this uncharted territory, revealing a noteworthy correlation between insurance coverage and the promptness of medical treatments [4]. This revelation has highlighted the potentially pivotal role of insurance status in shaping the timely delivery of healthcare services [7]. Yet, despite these significant findings, the scholarly landscape continues to grapple with a palpable gap in comprehensive inquiry into the intricate interplay between physicians’ perceptions and their navigation of the intricate disparities within the Saudi Arabian healthcare fabric.

The realm of healthcare delivery is inextricably intertwined with the influence of physicians’ perceptions upon their clinical practices [8]. Kruk et al. have illuminated the pivotal role of physicians’ understanding of the healthcare system, particularly insurance policies, in shaping their clinical decisions [9]. The healthcare landscape of Saudi Arabia elegantly harmonizes traditional values and progressive medical practices, and beckons for an in-depth exploration into how physicians’ perceptions meld with the intricate nuances of insurance status. This interplay signifies a critical determinant that can potentially shape the trajectory of medical choices. To illuminate this complex interplay, it is essential to navigate the multifaceted cultural and healthcare framework within which Saudi Arabian physicians operate [10]. Understanding these perceptions within this broader context becomes a cornerstone for unraveling the complexities that underline the intricate interplay between insurance status and physicians’ medical decisions.

Saudi Arabia’s societal fabric intricately weaves together cultural, social, and economic threads that can potentially overlay the interface between insurance status and clinical decisions [11]. The scholarly insight provided by Alodhayani et al. has highlighted the discernible impact of cultural norms and societal expectations on medical choices [12]. This multifaceted interjection necessitates a deliberate exploration of how these cultural and contextual elements intersect with insurance-related considerations. This intersection amplifies the intricate dynamics that influence physicians’ decision-making processes, accentuating the complexity of their responses to insurance-related factors. Unpacking these multidimensional layers provides a more profound understanding of the intricate influences that shape clinical choices within the dynamic Saudi Arabian healthcare landscape.

Hence, this study aimed to investigate how physicians perceived the influence of insurance status on treatment options and medical interventions and to explore the extent to which physicians discussed insurance-related considerations with patients during the shared decision-making process.

## Materials and methods

Study design and area

This research employed a cross-sectional exploratory study conducted in various healthcare facilities all over Saudi Arabia from April to October 2023. The study involved data collection regarding their perceptions of the impact of insurance status on clinical decision-making.

Participants' inclusion and exclusion criteria

The study participants included practicing physicians from various specialties and categories (consultants, registrars, senior registrars/general practitioners) working in private or both types of practices (government and private) and holding Saudi and non-Saudi nationality. Doctors exclusively working in government settings were excluded.

Data collection

Data were collected using an electronic questionnaire. The questionnaire was composed of two sections, including sociodemographic information and the Modified 9-item Shared Decision-Making Questionnaire (SDM-Q-Doc, physician version) [13]. SDM-Q-Doc was modified to reflect the context of insurance-related considerations in clinical decision-making.

Data sampling

A stratified random sampling technique was used to collect data.

Data analysis

Data collected through the questionnaire was analyzed using both descriptive and inferential statistical methods using IBM SPSS Statistics for Windows, Version 23.0 (IBM Corp., Armonk, NY) software. Descriptive statistics were used to summarize sociodemographic information and participants' responses to individual questionnaire items. Inferential statistics such as analysis of variance (ANOVA) and student's t-test were employed to identify significant associations between participants' characteristics and their perceptions of insurance-related influences on clinical decision-making. *P*-value was considered significant if it was less than 0.05.

Ethical considerations

Ethical approval was sought from an appropriate institutional review board (IRB) before conducting the study. Informed consent was obtained from all participating physicians. To ensure confidentiality, all collected data were anonymized and stored securely. This study was a non-interventional, survey-based research focusing on the impact of insurance status on physicians' clinical decision-making processes. The study methodology did not involve direct patient interactions, use of human tissue, or access to identifiable patient records. Furthermore, all data collected were entirely anonymous, ensuring that no identifiable information was used or mentioned.

## Results

Participants' characteristics

About 430 participants were included in the current study, of which 230 (53.5%) were male and 200 (46.5%) were female. Half of the participants (*n *= 215, 50%) were within the age group of 31 to 40 years. About 145 (33.7%) were Saudi, and the rest 285 (66.3%) were non-Saudi. The majority of the participants (*n *= 258, 60%) were medical officers with one to five years of experience (*n *=187, 43.5%). Three hundred one participants (70%) were engaged in private practice only, while the remaining 129 participants (30%) were involved in both private and governmental practices (Table 1).

**Table 1:** Sociodemographic characteristics of the study participants (n = 430). The data have been represented as *n* and %.

Variable	Categories	Frequency	Percentage (%)
Gender	Male	230	53.5
	Female	200	46.5
Age (in years)	25-30	71	16.5
	31-40	215	50
	41-59	116	27
	60 or more	28	6.5
Nationality	Saudi	145	33.7
	Non-Saudi	285	66.3
Category	General practitioner	115	26.7
	Registrar	101	23.5
	Senior registrar	100	23.3
	Consultant	114	26.5
Specialty	Dentist	71	16.5
	Medical	258	60
	Surgical	101	23.5
Years of experience	Less than one year	15	3.5
	1-5 years	187	43.5
	6-10 years	156	36.3
	More than 10 years	72	16.7
Type of practice	Private-only	301	70
	Both (Government/Private)	129	30

Physicians’ perceptions of insurance status impact on clinical decision-making

About 287 (66.7%) participants agreed that they take into consideration the patient's insurance status when discussing potential treatment options. Around 317 (73.7%) agreed that they would ensure that their patients are aware of how their insurance status might impact the range of treatment options available to them. Discussing the financial implications of different treatment options with patients considering their insurance coverage was agreed by 318 (74%) participants. Differences in the treatment choices made by patients based on their insurance status and insurance-related considerations influenced the time was agreed by 331 (77%) and 288 (67%) participants, respectively. Almost 373 (86.7%) participants agreed that insurance status can impact patient outcomes and treatment effectiveness, and 274 (63.7%) participants agreed that insurance-related factors played a role in the shared decision-making process between them and their physicians. However, 260 (60.5%) felt that patients with different insurance statuses received equitable medical care. Only 318 (74%) participants agreed that addressing insurance-related disparities in healthcare is essential for improving patient outcomes (Table 2).

**Table 2:** Physicians’ perceptions of insurance status impact on clinical decision-making. The data have been represented as *n* and %.

Statement	Disagree, *n* (%)	Neutral, *n* (%)	Agree, *n* (%)
1. I take into consideration a patient's insurance status when discussing potential treatment options.	56 (13)	87 (20.2)	287 (66.7)
2. I ensure that my patients are aware of how their insurance status might impact the range of treatment options available to them.	14 (3.3)	99 (23)	317 (73.7)
3. I discuss the financial implications of different treatment options with patients, considering their insurance coverage.	0 (0)	112 (26)	318 (74)
4. I have observed differences in the treatment choices made by patients based on their insurance status.	0 (0)	99 (23)	331 (77)
5. Insurance-related considerations influence the time taken by patients to receive necessary medical interventions.	42 (9.8)	100 (23.3)	288 (67)
6. I believe that insurance status can impact patient outcomes and treatment effectiveness.	28 (6.5)	29 (6.7)	373 (86.7)
7. Insurance-related factors play a role in the shared decision-making process between me and my patients.	0 (0)	156 (36.3)	274 (63.7)
8. I feel that patients with different insurance statuses receive equitable medical care.	29 (6.7)	141 (32.8)	260 (60.5)
9. I believe that addressing insurance-related disparities in healthcare is essential for improving patient outcomes.	28 (6.5)	84 (19.5)	318 (74)

**Figure 1:**
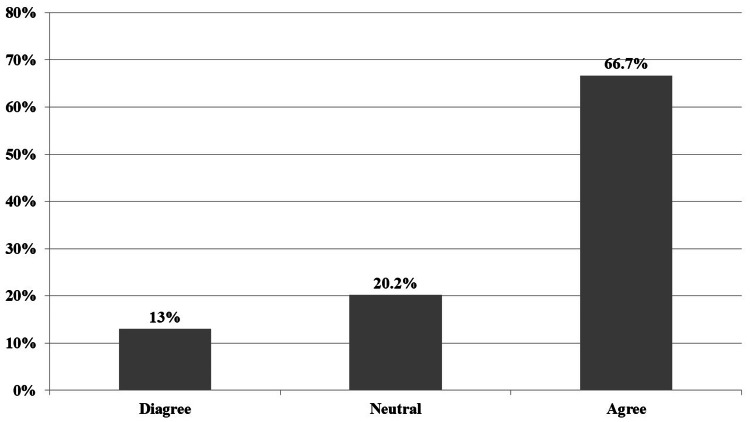
Physicians' answers about whether they take into consideration a patient's insurance status when discussing potential treatment options.

When discussing treatment options, 287 (66.7%) participants agreed that they considered the patient's insurance status (Figure 1).

The mean of the SDM-Q-Doc total score was found to be 24.0 ± 3.21 (range 13-27) out of a total of 27. A statistically significant association was found between years of experience and the score of insurance status impact on clinical decision-making (*P *= 0.002). Participants with more than 10 years of experience had a higher score of insurance status impact on clinical decision-making. Physicians in the age group of 31 to 40 years had a higher score of insurance status impact on clinical decision-making (*P* < 0.001 and 0.005, respectively). The type of practice was also found to be significantly associated with the score of insurance status impact on clinical decision-making (*P* = 0.012). Participants practicing in both private and governmental sectors had a higher score of insurance status impact on clinical decision-making (Table 3).

**Table 3:** Factors associated with physicians' perceptions of how insurance status impacts clinical decision-making. The data have been represented as mean ± SD. A higher mean reflects a higher impact on clinical decision-making. *P*-value was considered significant at *P *< 0.05.

Variable	Categories	Score of insurance status Impacts on clinical decision-making		*P*-value
		Mean	SD	
Gender	Male	23.85	3.684	0.381
	Female	24.12	2.555	
Age (in years)	25-30	23.00	3.740	<0.001
	31-40	25.38	2.113	
	41-59	24.83	2.242	
	60 or more	23.50	1.528	
Nationality	Saudi	24.06	2.852	0.710
	Non-Saudi	23.93	3.379	
Category	General practitioner	24.16	2.664	0.382
	Registrar	24.33	2.367	
	Senior registrar	23.75	2.717	
	Consultant	23.68	4.495	
Specialty	Dentist	24.24	2.659	0.005
	Medical	24.26	3.402	
	Surgical	23.07	2.892	
Years of experience	Less than 1 year	25.00	0.000	0.002
	1-5 years	23.43	3.521	
	6-10 years	24.04	3.364	
	More than 10 years	25.04	1.682	
Type of practice	Private only	23.76	3.528	0.012
	Both (Government/Private)	24.47	2.226	

## Discussion

Determination of physicians’ perception about how insurance status impacts clinical decision-making. It also plays an undeniable role in deciding the patient's plan of management to be finalized from the patient's side [3].

About two-thirds (287, 66.7%) of participants agreed that they considered the patient's insurance status when discussing potential treatment options; similar findings were reported in a congruent study [14].

More than two-thirds (317, 73.7%) agreed that they would ensure that their patients were aware of how their insurance status might impact the range of treatment options available to them; analogous findings were reported in Murray's study [15].

Discussing the financial implications of different treatment options with patients considering their insurance coverage was agreed by 318 (74%) participants; similar findings were mentioned in a parallel study in which decision-making belonged to both the physician and the patient [16]. More than two-thirds (318, 74%) of the participants agreed that addressing insurance-related disparities in healthcare is essential for improving patient outcomes. And 373 (86.7%) participants agreed that insurance status can impact patient outcomes and treatment effectiveness.

Out of 27, the mean SDM-Q-Doc total score was found to be 24.0 ± 3.21, whereas similar findings were reported in a congruent study [17].

Regarding the association between factors related to physicians’ perception of how insurance status impacted clinical decision-making; age was found to be significantly associated with the score as participants aged 31-40 years had higher scores than others, while contradictory findings were reported in the study [17]. A statistically significant association was found between years of experience and score of insurance status impact on clinical decision-making, with participants who had more than 10 years of experience having the higher score. The type of practice was also found to be significantly associated with the score of insurance status impact on clinical decision-making. Similar results were reported in a congruent study, which demonstrated that the type of practice influenced the physicians’ clinical decision-making in patients with insurance [18,19].

At the heart of modern healthcare ecosystems lies the instrumental role of Health Information Systems (HIS) in optimizing care delivery. Patient information, harvested meticulously from these systems, informs clinical decisions while potentially recalibrating the weight assigned to insurance-related factors. The investigation in some prior studies aptly emphasized the precision of diagnoses and treatment strategies catalyzed by the integration of comprehensive electronic health records [20, 21]. However, within the unique contours of the Saudi Arabian healthcare milieu, the intricate dance between HIS integration and the delicate balance of insurance status and clinical decisions remains an untrodden path. Delving into this domain holds the promise of unearthing the extent to which technology-driven insights reshape physicians’ perceptions regarding treatment decisions, subsequently finding expression in the intricate tapestry of clinical scenarios [22]. This exploration extends beyond mere technological integration, unveiling the dynamic ways in which healthcare systems evolve to harmonize diverse determinants, including insurance status, into the delicate fabric of clinical decision-making processes.

This study had some limitations. Despite using stratified random sampling, there was insufficient randomization due to nonresponse from the section of the targeted sample to the online questionnaire resulting in the observed demographic characteristics of the current study sample as well as the subsequent result. Additionally, the exclusion of doctors who work only in government is devoid of the study of important sections and might affect the observed responses.

## Conclusions

This study concluded that insurance status’ had impact on the clinical decision-making process, which was influenced by specific parameters of physicians like age, experience, specialty, and type of practice. Physician-patient relationship and interaction should be improved and all management options should be discussed with the advantages and disadvantages of particular management option compared to other options to set the suitable plan of management for particular patients according to their economic and insurance status with the most proper management plan available and acceptable for the patient.
